# Selectivity of Copper by Amine-Based Ion Recognition Polymer Adsorbent with Different Aliphatic Amines

**DOI:** 10.3390/polym11121994

**Published:** 2019-12-02

**Authors:** Nor Azillah Fatimah Othman, Sarala Selambakkannu, Tuan Amran Tuan Abdullah, Hiroyuki Hoshina, Suchinda Sattayaporn, Noriaki Seko

**Affiliations:** 1 Radiation Processing Technology Division, Malaysian Nuclear Agency, Bangi, Kajang 43000, Selangor, Malaysia; azillah@nm.gov.my (N.A.F.O.); sarala@nm.gov.my (S.S.); 2 Centre of Hydrogen Energy, Institute of Future Energy, Universiti Teknologi Malaysia, Johor Bahru 81310, Johor, Malaysia; tamran@cheme.utm.my; 3 Takasaki Advanced Radiation Research Institute, Quantum Beam Science Research Directorate, 1233, Watanuki-machi, Takasaki 370-1292, Gunma, Japan; hoshina.hiroyuki@qst.go.jp; 4 Synchrotron Light Research Institute, Nakhon Ratchasima 30000, Thailand; suchinda@slri.or.th

**Keywords:** pre-radiation grafting, chemical functionalization, aliphatic amine, radiation crosslinking

## Abstract

This paper investigates the selectivity of GMA-based-non-woven fabrics adsorbent towards copper ion (Cu) functionalized with several aliphatic amines. The aliphatic amines used in this study were ethylenediamine (EDA), diethylenetriamine (DETA), triethylenetetramine (TETA), and tetraethylenepentamine (TEPA). The non-woven polyethylene/polypropylene fabrics (NWF) were grafted with glycidyl methacrylate (GMA) via pre-radiation grafting technique, followed by chemical functionalization with the aliphatic amine. To prepare the ion recognition polymer (IRP), the functionalized amine GMA-grafted-NWF sample was subjected to radiation crosslinking process along with the crosslinking agent, divinylbenzene (DVB), in the presence of Cu ion as a template in the matrix of the adsorbent. Functionalization with different aliphatic amine was carried out at different amine concentrations, grafting yield, reaction temperature, and reaction time to study the effect of different aliphatic amine onto amine density yield. At a concentration of 50% of amine and 50% of isopropanol, EDA, DETA, TETA, and TEPA had attained amine density around 5.12, 4.06, 3.04, and 2.56 mmol/g-ad, respectively. The amine density yield decreases further as the aliphatic amine chain grows longer. The experimental condition for amine functionalization process was fixed at 70% amine, 30% isopropanol, 60 °C for grafting temperature, and 2 h of grafting time for attaining 100% of grafting yield (Dg). The prepared adsorbents were characterized comprehensively in terms of structural and morphology with multiple analytical tools. An adsorptive removal and selectivity of Cu ion by the prepared adsorbent was investigated in a binary metal ion system. The IRP samples with a functional precursor of EDA, the smallest aliphatic amine had given the higher adsorption capacity and selectivity towards Cu ion. The selectivity of IRP samples reduces as the aliphatic amine chain grows longer, EDA to TEPA. However, IRP samples still exhibited remarkably higher selectivity in comparison to the amine immobilized GMA-*g*-NWF at similar adsorption experimental conditions. This observation indicates that IRP samples possess higher selectivity after incorporation of the ion recognition imprint technique via the radiation crosslinking process.

## 1. Introduction

Copper is one of the unique elements in a heavy metal cluster which is classified as biogenic. Copper is vital in maintaining human health but only at minimum quantity, while excessive exposure is deadly carcinogenic. According to the World Health Organization (WHO), the permitted limit of Cu in water is 2.0 mg/L [1]. Industries such as fertilizers, motor vehicles, petroleum refineries, aircraft plating, steelwork foundries, and pulp and paper mills are typical contributors of Cu into the environment [2]. Thus, the removal of Cu from water is most vital. Commercially, numerous chemical and physical technological solutions are available for the removal of heavy metals such as Cu from water. Synthetic and natural material-based adsorbents onto the removal of toxic heavy metals had gained some popularity in recent years [3].

Polymeric materials provide good quality of water, reusable, cost-effective, and easy to handle. Thus, polymeric materials own some advantages in comparison to other, which favors them to be used as an adsorbent. Anyhow, polymer requires surface modification to provide good adsorptive properties. One of the recognized techniques available for the polymeric material surface modification is radiation-induced graft polymerization [4]. Glycidyl methacrylate (GMA) was widely used as a precursor monomer, mostly for the surface modification of polymeric materials via the radiation-induced graft polymerization technique. It, as an epoxy group is susceptible to functionalization with various type functional groups [5]. Among all the functionalization precursor agents, amine had imposed strong chelating properties towards transition metals. Previously, numerous studies that applied substrates such as cellulose [6], polystyrene [7], polyethylene non-woven fabrics [4], polyglycidyl methacrylate (PGMA) [8], and composite materials [9]. These materials had been functionalized with various amines to prepare an adsorbent for the application of recovering heavy metal ions from aqueous solution [10,11,12].

Surface functionalization of polymer materials with PGMA was preferred in most of the studies, as PGMA exhibits unique properties. Since PGMA has the reactive epoxy group which can be easily modified by various functional groups, it could be introduced good mechanical strength, high acid-base resistance [5,8,13]. Amines such as introducing with EDA, DETA, and TETA has used to functionalize PGMA-based materials for the metal ions adsorption [14,15]. Numerous studies had been conducted to identify the relationship between the efficiency of metal ions removal with a molecular chain length of amines. Studies had explicitly been carried out to investigate the role of an amine with a longer molecular chain and amine group density on metal ion adsorption capacity [4,8].

Adsorbent incorporated with selective properties possesses a significant advantage over adsorption of targeted metal ion individually. Adsorbents with a nitrogen-based functional group are very effective in the removal of heavy metal ions [16]. Several techniques employed to associate selective removal characteristics onto adsorbent [8,17]. On top that, despite many studies reported on metal ion adsorption capacity varies on numerous amines, nothing was emphasized on the selectivity of metals ions based on the molecular chain length of amines. Thus, this study focused on the selectivity of metal ions by adsorbents functionalized with different aliphatic amines. In this study, selectivity characteristics were incorporated onto amine immobilized GMA-based polymer adsorbent via radiation crosslinking technique to form a cavity that is complementary to the targeted ion. The polymer adsorbent which denotes hereafter as ion recognition polymer (IRP) works based on preorganized functional monomer interaction with imprinted template ion via ascertaining chemical interface among them by the aid of the crosslinking process after that [18]. Recognition of printed ion is possible by the presence of specific binding sides onto IRP materials which supports the higher selectivity for the rebinding of printed ion.

In this study, the polymeric non-woven fabrics (NWF) grafted with GMA via radiation graft polymerization technique, followed by subsequent chemical functionalization with different aliphatic amines. The amine-functionalized GMA-*g*-NWFs were subjected to the radiation crosslinking process with divinylbenzene (DVB) for the preparation of ion recognition samples. Prior to the radiation crosslinking process, the amine-functionalized GMA-*g*-NWF subjected to adsorption of Cu ion, as the template for the preparation of the IRP sample. The IRP was designed in a way that resembles the unique fitting of a key to its corresponding lock. Subsequent removal of the template leaves binding sites cavities complementary in shape, size, and functional groups orientation in the polymer network. Finally, adsorption of Cu ions was carried out in binary metal ions system to examine the selectivity. The effect of different aliphatic amines on adsorption capacity and selectivity of the prepared IRP sample was investigated thoroughly.

## 2. Experimental

### 2.1. Materials

The polymer trunk, PE/PP NWF was obtained from Kurashiki Textile Manufacturing Co. Ltd. (Osaka, Japan). Monomer with 97% purity of GMA, and polyoxyethylene sorbitol ester (T-20) were provided by Tokyo Chemical Industry Co. (Tokyo, Japan). The amines at 95% purity of ethylenediamine (EDA), diethylenetriamine (DETA), triethylenetetramine (TETA), and tetraethylenepentamine (TEPA), were used for the chemical modifications that were purchased from Kanto Chemical Co. Inc. (Tokyo, Japan). Isopropanol as the solvent was purchased from Kanto Chemical Co. Inc. The copper ion solution for the adsorption test was prepared from 1000 ppm standard solutions provided by Merck Chemicals (Bandar Sunway, Malaysia). All chemicals were of analytical grade.

### 2.2. Methodology

#### 2.2.1. Preparation of Amine Immobilized GMA-g-NWF

**Figure 1:**
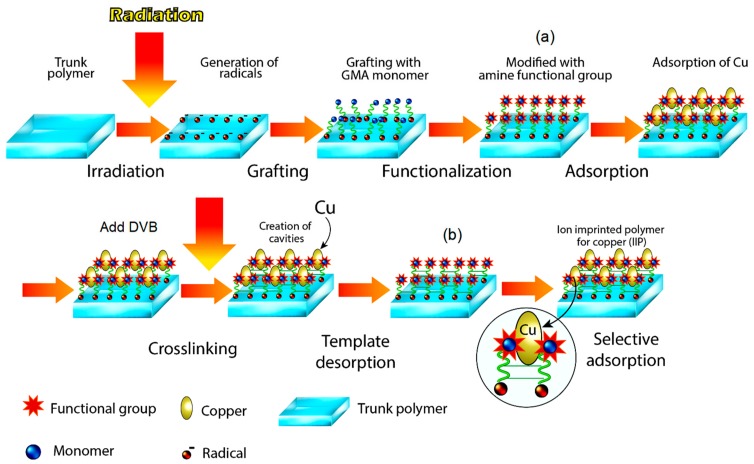
Preparation of (**a**) Amine immobilized GMA-g-NWF, and (**b**) Ion recognition polymer (IRP).

The amine immobilized GMA-*g*-NWF was prepared using a pre-radiation grafting technique which followed by subsequent chemical functionalization process with aliphatic amines. The complete scheme for preparation amine immobilized GMA-*g*-NWF shown in Figure 1a. Prior to functionalization with an amine, pre-irradiation grafting of GMA was carried out onto 0.5 g of NWF sample which was purged with nitrogen and seal in a polyethylene bag. Then, the samples embedded in the bag irradiated with a low energy electron beam accelerator at 250 keV energy, 1.8 mA and irradiation dose of 10 kGy/pass. Total irradiation dose received by NWF samples estimated at around 30 kGy. The emulsified 5 wt. % GMA with 0.5 wt. % T-20 solution transferred into ampoule which containing irradiated NWF samples. The ampoule was placed in a water bath at 40 °C for 1.0 h for the grafting process. Finally, the samples washed with distilled water and placed in an oven at 110 °C for overnight.

The pre-irradiation grafting process parameters were calculated to obtain total grafting yield (*D*_g_), was calculated as in Equation (1):

(1) $$D_{g}\left(\%\right)=\frac{W_{g}-W_{0}}{W_{0}}\times 100$$

whereas *W*_o_ and *W*_g_ are the initial and final weight of NWF before and after grafting. *D*_g_ obtained based on gravimetric measurement is commonly on practice and also proven to be comparable to other methods as well [19].

For the functionalization process, 0.5 g of GMA-*g*-NWF which had attained *D*_g_ about 100% was immersed in a mixture of amine and isopropanol solution in a total volume of 50 mL at several ratios. The different concentrations of amine in isopropanol were varied to investigate the effect of amine concentration on amine density (AD). Subsequently, the ampoule was placed in a water bath for a chemical reaction to take place in due course. The same procedure has adhered to all the aliphatic amines. The effect of *D*_g_, reaction temperature, and reaction time on AD in the presence of different aliphatic amines were studied as well. The AD was calculated according to Equation (2):

(2) $$AD=\frac{\left(Z_{1}-Z_{0}\right)/Z_{0}}{M}\times 1000$$

where *Z*_1_ and *Z*_0_ denote the weight of the GMA-g-NWF in (gram) after and before the functionalization process, and M is the molecular weight of amine in (gram/mole) [20].

All experiments were performed in duplicate, and all measurements were done using the same instruments for consistency.

#### 2.2.2. Preparation of Ion Recognition Polymer (IRP) Adsorbent

The amine immobilized GMA-g-NWF was further modified, which later acknowledged as IRP (Figure 1b) by irradiation crosslinking of DVB with a complex of amine immobilized GMA-*g*-NWF and copper (Cu) using high energy radiation to enhance the selective adsorptive properties of the polymer adsorbent further.

The amine immobilized GMA-g-NWF with about 100% grafting yield and AD approximately 2.0–2.2 mmol/g^−ad^ was subjected to a mutual irradiation-induced crosslinking process in the present of emulsified DVB solution. Prior to the radiation crosslinking, the amine immobilized GMA-*g*-NWF was subjected to adsorption of Cu ion (10 mg/L, pH 4.0, 3 h) in an aqueous medium. Upon completion of the adsorption process, the IRP was washed thoroughly with distilled water and dried in an oven overnight at 50 °C. After that, the sample was immersed in an emulsified crosslink solution of DVB/T-20 (2:1 ratio) in a polyethene bag and subsequently purged with nitrogen to remove dissolved oxygen. The sample is then exposed to a Co-60 gamma source a dose rate of 10 kGy/h. The sample was placed in a water bath at 60 °C for 2 h after completion irradiation process. Finally, the samples were washed comprehensively with distilled water and methanol and dried in an oven at 50 °C overnight. Then, the crosslinking percentage was calculated using ASTM D 2765-95 method.

The desorption of the adsorbed Cu from the IRP matrix sample was carried out by stirring it with 0.1 M of HNO_3_ for 2 h at room temperature. Finally, the sample was rinsed with distilled water and dried in an oven at 50 °C overnight before.

All experiments were performed in duplicate, and all measurements were done using the same instruments for consistency.

#### 2.2.3. Batch Adsorption Test

The adsorption process was conducted in the binary metal ion system which contains the mixture of Cu and lead (Pb) ion to validate the selectivity of the amine immobilized GMA-*g*-NWF and IRP samples towards Cu ion. Approximately 0.05 g of the prepared absorbent samples were tested by immersed into 50 mL of the solution with continuous stirring at a pH of 4 with 0.5 M HNO_3_. The reaction time was allocated up to 8 h to ensure equilibrium adsorption for amine immobilized GMA-*g*-NWF. The reaction time for IRP was set accordingly for comparison purpose. The residual metal in the solution was determined by Inductively Coupled Plasma–Optical Emission Spectrometry, ICP–OES (Thermo Fisher Scientific, iCAP 7200) (Waltham, MA, USA). The adsorption capacity (Q) was calculated via Equation (3).

(3) $$Q=\left(\frac{C_{0}-C_{e}}{1000m}\right)V$$

where C_0_ and C_e_ are the initial and equilibrium concentrations (mg/L) of metal ion solution and V (mL) is the volume of the metal ion solution, and *m* (g) is the mass of the amine immobilized GMA-*g*-NWF.

Moreover, the selectivity (*K*_s_) of the samples towards Cu was also appraised in the binary solution. was calculated by Equations (4) and (5). However, the Equation (4) requires the calculation of the distribution coefficient (*K*_d_), which define as Equation (5);

(4) $$K_{s}=\frac{K_{d}\left(Copper\right)}{K_{d}\left(Lead\right)}$$

(5) $$K_{d}=\left(\frac{C_{i}-C_{f}}{C_{f}}\right)\frac{V}{m}$$

where, *C*_i_ and *C*_f_ are the initial and final concentrations of metal ions (mg.L^−1^), V is the volume of solution (mL), and m is the mass of amine immobilized GMA-*g*-NWF (mg). *K*_d_(Copper) and K_d_(Lead) are the distribution coefficients of Cu and Pb in dimensionless units, respectively [21].

All experiments were performed in duplicate, and all measurements were done using the same instruments for consistency.

### 2.3. Structural and Morphological Confirmation

The surface morphology of the samples was imaged by field emission scanning electron microscope, FESEM (Zeiss, GeminiSem 500-70-22) (Karlsruhe, Germany) at 10.0 kV and 107.4 µA of voltage and current, correspondingly. The magnifications range of 200 times was used with a resolution of 20 nm.

The Fourier-transform infrared, FTIR (Bruker, Tensor II) (Karlsruhe, Germany) analysis was performed to identify the chemical functionalities present in the samples. The measurement was carried out in with single reflection diamond universal attenuated total reflection (ATR) mode scanned from 4000 to 400 cm^−1^ at a resolution of 4 cm^−1^.

Water uptake capacity of amine immobilized GMA-*g*-NWF, and IRP samples were tested by the gravimetric method. Approximately, 1.0 g of anticipated samples were immersed in 50 mL of distilled water at room temperature along with continuous stirring. The weight of the samples was measured at a fixed time interval until saturation was achieved. The percentage of water uptake is calculated using the following Equation (6);

(6) $$WUC\left(\%\right)=\frac{m_{2}-m_{1}}{m_{1}}\times 100\%$$

where *m*_1_ and *m*_2_ are the weight of the sample before and after water adsorption.

The adsorptive interaction between Cu ions in the IRP samples was observed under X-ray photoelectron spectroscopy, XPS (ULVAC-PHI, Quantera II) (Chigasaki, Japan) with an X-ray source: monochromatic Al-Kα (λ_v_ = 1486.6 eV), which operated at 25.6 W (beam diameter of 100 µm). Wide scan analysis was executed using a pass energy of 280 eV with 1 eV per step. A narrow scan was performed by pass energy of 112 eV with 0.1 eV per step.

X-ray absorption experiments were performed at BL 5.2 of the Synchrotron Light Research Institute (SLRI), Thailand to determine the ligand structural coordination before and after the radiation crosslinking process. The storage ring operates at 1.2 GeV and a typical current ranging from 80 to 150 mA. The beamline delivers X-rays with the maximum photon flux between 1.1–1.7 × 10^11^ photons·s^−1^ employing a bending magnet. The synchrotron radiation was monochromatized by a commercial double-crystal X-ray monochromatic which equipped with Si (111) crystals. X-ray absorption fine structure (XAFS) spectra were collected in transmission mode from 200 eV before the edge up to k = 13 with a spectral resolution of 0.03 k.

## 3. Results and Discussion

### 3.1. Functionalization of Aliphatic Amine

#### 3.1.1. Effect of Amine Concentration on Amine Density

The amine functionalization process was carried out at different concentrations of amine and isopropanol solution to investigate effect onto AD. The concentration of amine: isopropanol solution used was 50:50%, 60:40% and 70:30% by volume. The results attained exhibited in Table 1. As the concentration of aliphatic amine increases in the amine solution, from 50% to 70% (vol.), the AD had demonstrated a descending pattern. The density reduces from 5.12 to 3.80 mmol/g^−ad^ as the EDA concentration increases. Similar trends observed with DETA, TETA, and TEPA as well. As the concentration of aliphatic amine increases, the portion of isopropanol which had used as solvent reduces. It is known that solvent swells the polymer backbone, and therefore helps the functional group additive to reach the active sites [22]. Thus, the reduction of solvent fraction volume has a definite influence in the amine functionalization. This leads to a deceleration in the functionalization process which had shown lower AD yield.

On the other hand, the AD reduces as the concentration of EDA, DETA, TETA, and TEPA increases. At the same concentration of 50% of amine; 50% of isopropanol, EDA, DETA, TETA, and TEPA had attained AD around 5.12, 4.06, 3.04, and 2.56 mmol/g-ad, respectively. The functionalization process takes place by fusion of amine group onto poly-GMA chains upon the opening of the epoxy group of GMA [23]. Among all four amine EDA had shown a higher yield of AD and reduced further with an increase in the number of the amine group. The molecular weight and structure grow more significant as the number amine group increases as well as the aliphatic chain. This may attribute to the steric hindrance effect which prevents the fusion of the amine group onto poly-GMA chains [24]. Thus, it leads to lower AD yield as the EDA, DETA, TETA, and TEPA, molecular weight, and structure progress further.

#### 3.1.2. Effect of Grafting Yield on Amine Density

GMA-g-NWF with grafting yield of approximately 100%, 150%, 200% and 250% (experimental data are shown in Table 2) was used to obverse the trend in AD yield upon functionalization with multiple aliphatic amines. The AD increase as the grafting yield increases from 100% to 250% for EDA, DETA, TETA, and TEPA aliphatic amine. The EDA achieves the AD of 3.35 mmol/g^−ad^ with the grafting yield of 100%, which increases to 4.20 mmol/g^−ad^ while the grafting yield of GMA-*g*-NWF increases to 250%. The same trend was observed for DETA, TETA, and TEPA. This phenomenon well explained by the availability of additional epoxy groups from GMA at higher grafting yield for the incorporation of the amine group will favorably support the process to occur [25]. Thus, the AD increase constantly as the grafting yield increases. On the other hand, AD decreases steadily as the chains of the aliphatic amine grow longer from EDA to TEPA. A similar observation was obtained as the changes in grafting yield of GMA-*g*-NWF were made. The molecule size increases as the molecular chain length of aliphatic amine increasing from EDA to TEPA. This induced steric effect which eventually increases the inter-molecular repulsions caused by the additional chain length in the structure, preventing it from accessing and reacting with the epoxy groups of the GMA. A similar observation was witnessed by Liu et al. [11] where they experienced decreased in calculated nominal polyamine density as the chain length grows during the immobilization on grafted adsorbents.

#### 3.1.3. Effect of Reaction Temperature on Amine Density

The amine functionalization was carried out at 60 to 80 °C to study the effect on the AD. The effect of temperature was also applied to the EDA, DETA, TETA, and TEPA. The results attained exhibited in Table 3. The AD increases marginally as the reaction temperature increase from 60 to 80 °C for EDA, DETA, TETA, and TEPA aliphatic amine. The EDA achieve the AD of 3.85 mmol/g^−ad^ at 60 °C and increases to 3.93 mmol/g^−ad^ at 80 °C. A similar trend was observed with DETA, TETA, and TEPA as well. The thermal energy accelerates the reaction at a high temperature above 60 °C, which leads to an increased migration rate of the amine group onto the epoxy group of GMA [26]. In contrast, AD gradually reduces as the chains of the aliphatic amine grow longer from EDA to TEPA at the same course temperature. At reaction temperature of 60 °C, AD of 3.85, 3.31, 2.97, and 2.49 mmol.g^−ad^ were attained for functionalization with EDA, DETA, TETA, and TEPA aliphatic amine, respectively. This agrees well with the previous observation at different experimental conditions. In addition, since the introduced amine groups were polymerized, no significant decomposition was observed in the high-temperature reaction. Conclusively, the bulky structure of aliphatic amine restrains efficiency of the functionalization predominantly regardless of any of the changes in experimental conditions. Thus, the higher AD could be achieved by using the smallest and simplest aliphatic amine, EDA.

#### 3.1.4. Effect of Reaction Time onto Amine Density

The effect of reaction time had been analyzed on the assimilation reaction of amine functional group onto GMA-g-NWF. The reaction time was 15, 30, 45, 60, 90, and 120 min. The results achieved are shown in Table 4. The AD increases considerably with reaction time but reaches almost plateau around 30 min, and thereafter insignificant gain was observed in AD as the reaction increases further. Other amines show a similar trend. EDA had gained an AD of 2.89 mmol/g-ad at 15 min of reaction time, which increases to 3.80 mmol/g-ad around 30 min, but the final gain of AD recorded was around 3.91 mmol/g-ad after 120 min complete reaction course. Exhaustion of epoxy sites present on GMA-g-NWF is responsible for the insignificant gain in AD after 30 min [20]. Correspondingly, EDA had gained the highest AD which decreases uniformed as the aliphatic amine chains growth longer. The observation still sustained with the progress in reaction time. EDA, the most straightforward and smallest structure, showed a promising amine to attain high AD to produce effective adsorbent.

### 3.2. Characteristics of the Amine Immobilized GMA-g-NWF and IRP

#### 3.2.1. Surface Morphological Analysis

**Figure 2:**
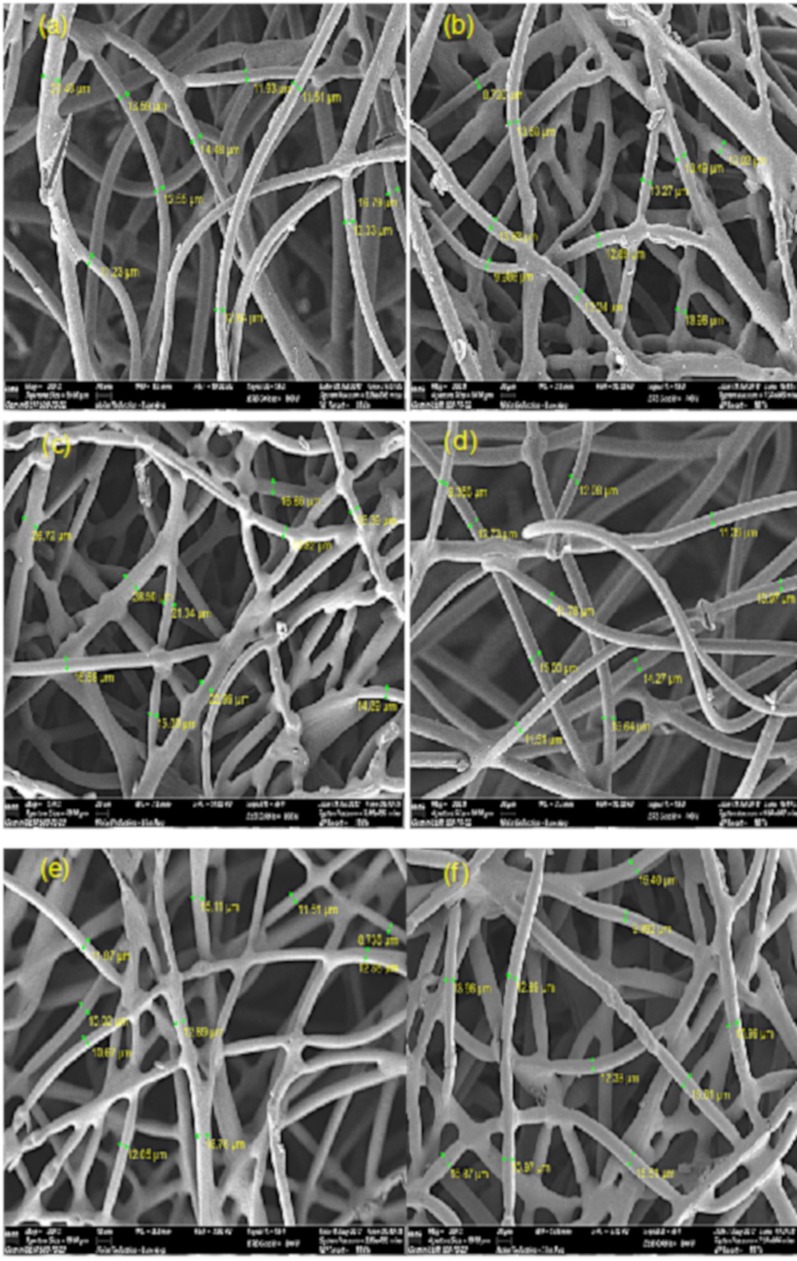

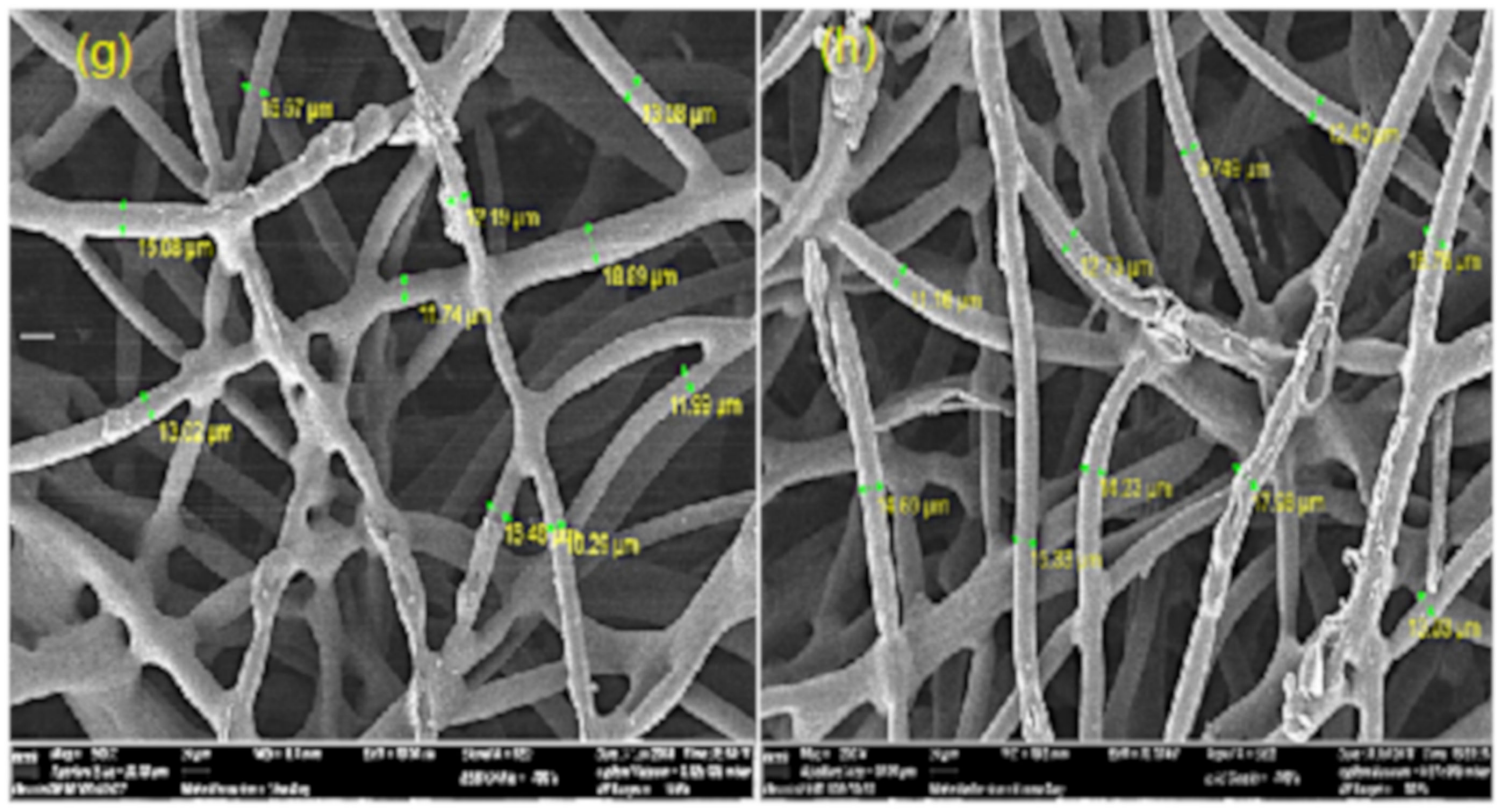
FESEM imaging of (**a**) EDA-GMA-*g*-NWF, (**b**) EDA-IRP, (**c**) DETA-GMA-*g*-NWF, (**d**) DETA-IRP, (**e**) TETA-GMA-*g*-NWF, (**f**) TETA-IRP, (**g**) TEPA-GMA-*g*-NWF and (**h**) TEPA-IRP at experimental condition of 70% amine: 30% isopropanol, 60 °C, *D*_g_ ~100% and 2 h.

Imaging onto the amine immobilized GMA-*g*-NWF and IRP samples that functionalized with the amines were carried out in order to observe the changes in terms of morphology before and after crosslinking process, respectively. The images obtained shown in Figure 2. The average diameter of the pristine sample was around 12.42 µm. The GMA-*g*-NWF sample obviously increases almost up to 13.44 µm. The increase in the diameter of the pristine sample after the grafting was due to the coating of the poly-GMA thin layer [27]. Moreover, the average diameter of GMA-*g*-NWF samples increases further after functionalization with a different aliphatic amine. The average diameter obtained for EDA-GMA-*g*-NWF, DETA-GMA-*g*-NWF, TETA-GMA-*g*-NWF, and TEPA-GMA-*g*-NWF was around 14.07, 18.91, 13.10 and 13.83 µm, respectively. This indicates the incorporation of the amine group on the epoxy group of GMA via the functionalization process [25]. Among four types of an aliphatic amine, DETA had shown the highest gain in term average diameter. On the other hand, after the crosslinking process, all the IRP samples a slight reduction in average diameter in comparison to amine immobilized GMA-*g*-NWF. The average diameter obtained for EDA-IRP, DETA-IRP, TETA-IRP, and TEPA-IRP was around 12.45, 12.48, 13.06 and 13.71 µm, respectively. Typically, when the polymer is subjected to radiation, two types of reaction, namely crosslinking and degradation, will occur [28]. The polymers mutually shrunk due to bonding of the polymer chains in the initial stage of radiation crosslinking, resulting from decreasing in average diameter after the crosslinking process. The differences in average diameter EDA-IRP, DETA-IRP, TETA-IRP, and TEPA-IRP were insignificant. Moreover, enormous weight loss also was not observed after radiation treatment which indicates that no occurrence of chain scission during the process.

#### 3.2.2. Elemental Composition Analysis

**Figure 3:**
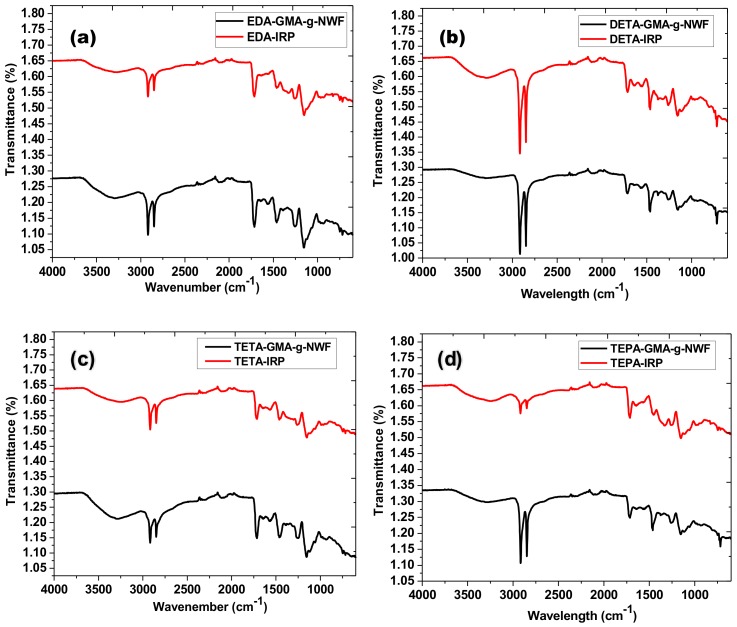
FTIR spectra of (**a**) EDA-GMA-*g*-NWF and EDA-IRP, (**b**) DETA-GMA-*g*-NWF and DETA-IRP, (**c**) TETA-GMA-*g*-NWF and TETA-IRP, (**d**) TEPA-GMA-*g*-NWF and TEPA-IRP at experimental condition of 70% amine: 30% isopropanol, 60 °C, Dg ~100% and 2 h.

The FTIR result of the immobilized amine samples was presented in Figure 3. Both GMA-g-NWF and IRP samples exhibits common stretching for polyethylene/polypropylene (PE/PP) at 2913, 2843, 1464, 1370 and 718 cm^−1^ which attributed to C–H stretch (PP), C–H stretch (PE), CH_2_ stretch (PP & PE), symmetric CH_3_ deformation stretch (PP) and CH_2_ stretch (PE) respectively, as similar described in other researcher [29]. Additional stretching detected at 1732 and 1140 cm^−1^ assigned for C=O and C–O vibration which originates from –COO– ester group of GMA [30]. However, additional stretching was found such as N–H stretching at 3201 cm^−1^, N-H bending at 1524 cm^−1^, C–N alkyl group stretching at 1256 and 1156 cm^−1^ which attributed from aliphatic amine groups [25]. Besides that, additional peaks at 1405, 1014 and 989 cm^−1^ which presents onto IRP samples attributed to C–H stretch. These are typical stretching vibration band of the vinyl groups which associated with in-plane and out-of-plane deformation bands of the vinyl group [31]. This peaks specifically attributed to the crosslinking process with DVB via radiation [32].

#### 3.2.3. Water Absorptive Capacity Analysis

**Figure 4:**
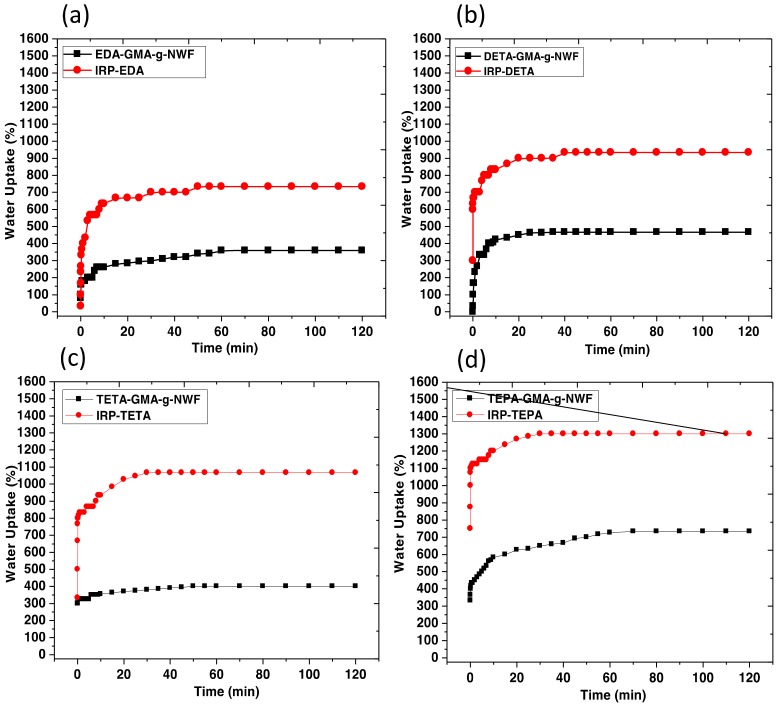
Water uptake capacity of (**a**) EDA-GMA-*g*-NWF and EDA-IRP, (**b**) DETA-GMA-*g*-NWF and DETA-IRP, (**c**) TETA-GMA-*g*-NWF and TETA-IRP, (**d**) TEPA-GMA-*g*-NWF and TEPA-IRP at experimental condition of 70% amine: 30% isopropanol, 60 °C, *D*_g_ ~100% and 2 h.

An adsorbent with good water uptake capacity is one of the important criteria as maximum delivery of water plays a crucial role in both adsorption and desorption processes in terms of energy efficacy [33]. Hence, the water uptake capacity of both amine immobilized GMA-*g*-NWF, and IRP samples were tested. The results obtained illustrated in Figure 4. Good cycling performance of an adsorbent is highly dependent on material’s water stability properties as well. Water uptake capacity of amine immobilized GMA-*g*-NWF increases as the aliphatic amine chains grow longer. The maximum water uptake capacity recorded for EDA-GMA-*g*-NWF, DETA-GMA-*g*-NWF, TETA-GMA-*g*-NWF, and TEPA-GMA-*g*-NWF samples were around 360%, 466%, 400% and 733%, respectively. A similar trend was attained for IRP samples. The maximum water uptake capacity recorded for EDA-IRP, DETA-IRP, TETA-IRP, and TEPA-IRP samples were around 733%, 933%, 1066% and 1300%, respectively. However, it is very noticeable that IRP samples exhibited higher water uptake capacity in comparison to amine immobilized GMA-*g*-NWF samples. One of the possibilities behind this is due to the morphology of the polymer that strongly affects the water uptake kinetics. For IRP samples, the imprinting effect generated an organized structure and enabled water to transport through the voids from the cavity.

### 3.3. Metal Ions Adsorption Performance

#### 3.3.1. Adsorption Capacity of Amine Immobilized GMA-*g*-NWF and IRP Samples Towards Cu

**Figure 5:**
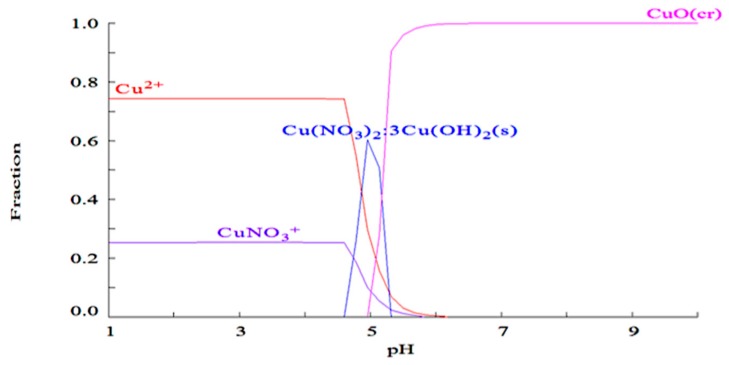
Expected speciation distribution of copper (10 mg/L) as a function of pH in nitric acid.

Amine immobilized GMA-*g*-NWF, and IRP samples were subjected to adsorption of a mixed solution of Cu and Pb ion at pH 4. The distribution of Cu species in aqueous was calculated Medusa and Hydra program [34] and illustrated in Figure 5. It can be seen from the figure that Cu^2+^ species was predominant up to pH 4.5. However, beyond pH 4.5, complexes started to occur and CuO complexes dominated with 100% total concentration at pH 5.5. Therefore, pH 4 was selected for all adsorption experiments in this study.

**Figure 6:**
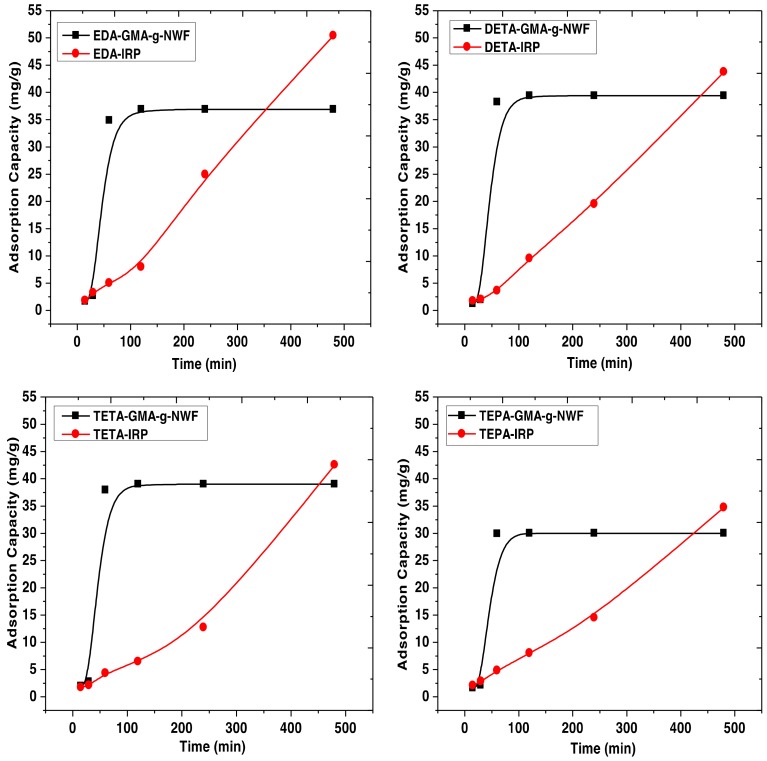
The adsorption capacity of Cu versus time by amine immobilized GMA-*g*-NWF and IRP samples at experimental condition of initial Cu/Pb concentration: 10 mg/L, adsorbent dose: 0.02 g, temperature: 30 °C, amine density: 2.0–2.2 mmol/g^-ad^, stirring speed: 200 rpm and adsorbate volume: 100 mL.

The adsorption capacity of both type samples was investigated. The result obtained for Cu adsorption is shown in Figure 6. The complete experimental data for adsorption capacity of both Cu and Pb is provided in the Supplementary Materials. The experimental conditions of the amine functionalization process were predetermined to attain AD approximately 2.0–2.2 mmol/g^−ad^ provided standardized condition was applied for adsorption experiment. The adsorption capacity of Cu for both amines immobilized GMA-*g*-NWF, and IRP samples were measured against adsorption time. The adsorption capacity of EDA-GMA-*g*-NWF towards Cu ion increases significantly for the initial 60 min, but after that, the adsorption capacity reaches almost plateau. The adsorption capacity EDA-GMA-*g*-NWF at first 15 min of adsorption was around 1.60 mg/g which increases up to 34.85 mg/g after 60 min of the process. The active sites present onto EDA-GMA-*g*-NWF more likely actively occupied during the initial stage of the adsorption process. However, subsequent saturation in adsorption of Cu ion merely takes place due to the exhaustion of active sites onto EDA-GMA-*g*-NWF [35]. The saturation is resulted from the simultaneous adsorption of Pb, suggesting possible competitive effect towards the active sites between Cu and Pb. A similar trend was attained for DETA-GMA-*g*-NWF, TETA-GMA-*g*-NWF, and TEPA-GMA-*g*-NWF samples as well. The adsorption capacity of amine immobilized GMA-*g*-NWF samples decreases as the aliphatic amines chain grows greater. The adsorption capacity towards Cu ion reduces with a trend of EDA-GMA-*g*-NWF < DETA-GMA-*g*-NWF< TETA-GMA-*g*-NWF < TEPA-GMA-*g*-NWF. These findings strongly related to the size of aliphatic amines. Since the EDA possesses the smallest size, thus the migration of EDA onto epoxy groups of GMA takes place in abundant in comparison to other aliphatic amines [4]. Another main reason for the reduction of adsorption capacity towards Cu ion as the number of amines increased from 2 to 5 is related to the covalent interactions relative to the ionic interactions during the competitive adsorption between Cu and Pb. Wang F. et al. [36] discussed the effect of the covalent index in their study, and they found out that Pb has a larger covalent index of 6.41 than Cu (2.64), which resulted in Pb having stronger interaction to the nitrogen and oxygen atoms to form complexes than Cu. This explained the reason of reduction in adsorption capacity towards Cu as aliphatic amines chain grows greater.

However, all the IRP samples had shown a different trend from amine immobilized GMA-*g*-NWF on adsorption capacity with time. The EDA-IRP sample had shown ascending trend with time for adsorption capacity of Cu ion. The adsorption capacity increases from 1.82 to 50.41 mg/g while the adsorption time increases from 15 to 480 min, respectively. Although the adsorption reaches the maximum allocated time of 480 min, the adsorption capacity of the EDA-IRP sample still did not show any sign for saturation. The EDA-IRP samples initially exhibit a slower adsorption rate. Since the adsorbent did not reach saturation, it is anticipated that there are more available active sites for Cu, which leads to higher Cu ion intake capacity. Due to the slow adsorption rate, the IRP does not reach a plateau as consequences of insufficient contact time between the solute and the adsorbent. The three-dimensional network formed through cross-linked imprinted polymeric material enables specific sites for designated ions which gives higher adsorption capacity at a slower adsorption rate [37]. A similar trend was attained for DETA-IRP, TETA-IRP, and TEPA-IRP samples as well. Meanwhile, IRP samples also illustrated a descending trend for adsorption capacity with aliphatic amines chain growth. The adsorption capacity towards Cu ion reduces with the trend of EDA-IRP < DETA-IRP < TETA-IRP < TEPA-IRP. The size of aliphatic amine plays a major role in the adsorption performance of both amines immobilized GMA-*g*-NWF and IRP samples.

#### 3.3.2. Selectivity of Amine Immobilized GMA-*g*-NWF and IRP Samples in Binary Metal Species System

**Figure 7:**
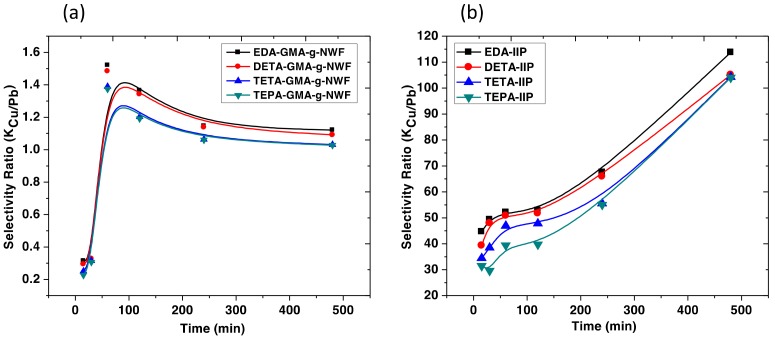
Selectivity ratio of amine immobilized (**a**) GMA-*g*-NWF and (**b**) IRP samples versus time at experimental condition of initial Cu concentration: 10 mg/L, adsorbent dose: 0.02 g, temperature: 30 °C, amine density: 2.0–2.2 mmol/g-ad, stirring speed: 200 rpm and adsorbate volume: 100 mL.

Both amines immobilized GMA-*g*-NWF, and IRP samples were subjected to selectivity experiment in binary metals system which consists of both Cu and leads ions. The main aim of the study is to investigate the selectively of the prepared polymeric material which enacted with the ion recognition technique in terms of several different aliphatic amines. The experimental conditions of the amine functionalization process were predetermined to attain AD approximately 2.0–2.2 mmol/g^−ad^ as a standardized condition when to apply for the selectivity experiment. The selectivity of both amines immobilized GMA-*g*-NWF and IRP samples towards Cu ions were measured against time, as shown in Figure 7. The selectivity of EDA-GMA-*g*-NWF towards Cu ion increases slightly for at the beginning of 60 min, then, the adsorption capacity reaches a plateau. However, the selectivity attained for EDA-GMA-*g*-NWF was only around 1.12. DETA-GMA-*g*-NWF, TETA-GMA-*g*-NWF, and TEPA-GMA-*g*-NWF samples, were also exhibited a similar trend. Therefore, all the amine immobilized GMA-*g*-NWF had shown insignificant selectivity towards Cu ion. It can be concluded that EDA-GMA-g-NWF, DETA-GMA-*g*-NWF, TETA-GMA-*g*-NWF, and TEPA-GMA-*g*-NWF samples possess good adsorption capacity for Cu ions in a single metal ion system and own very poor selectivity towards Cu ion in the binary metal system.

However, the EDA-IRP sample exhibited excellent selectivity towards Cu ion in the binary metal system. The selectivity of EDA-IRP increases from 44% to 113% while the adsorption time increases from 15 to 480 min, respectively. The selectivity of the EDA-IRP sample unable to reach the plateau even after 480 min of the adsorption process. This may due to the multilayer adsorption process, which requires a longer time to reach the equilibrium. This may further be confirmed by sorption isotherm data [38] in a future study. This observation is complimentary with the adsorption capacity of EDA-IRP towards Cu. DETA-GMA-*g*-NWF, TETA-GMA-*g*-NWF, and TEPA-GMA-*g*-NWF samples showed similar outcomes as well. The IRP samples own some cavities which are complimentary to imprinted Cu ion in terms of size and coordination geometry. Meanwhile, amine immobilized GMA-*g*-NWF shows a random distribution of functionalities in the polymeric network without any rebinding affinities specificity [39]. Therefore, amine immobilized GMA-*g*-NWF randomly adsorb any metal ions which preset in the absorption system without being selective towards Cu. The improvement on selectivity is limited as the equilibrium state was not achieved within the experimental duration. However, the selectivity still shows improvement almost 100 times by ratio. This is considered to be very good which align with previous findings [37]. The selectivity of IRP samples decreases as the aliphatic amines chain grows greater. The selectivity towards Cu ion reduces slightly with a trend of EDA-GMA-*g*-NWF< DETA-GMA-*g*-NWF< TETA-GMA-*g*-NWF< TEPA-GMA-*g*-NWF. The chain length and size of aliphatic amine plays important factor in this. Complex structural coordination with longer aliphatic amine chains may inhibit diffusion of Cu ions into template cavities.

#### 3.3.3. Adsorption Mechanism

**Figure 8:**
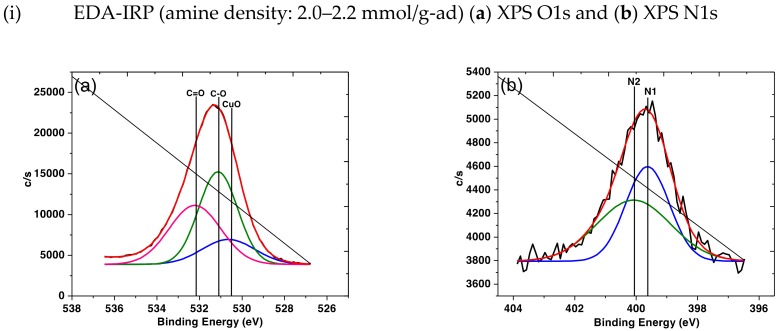

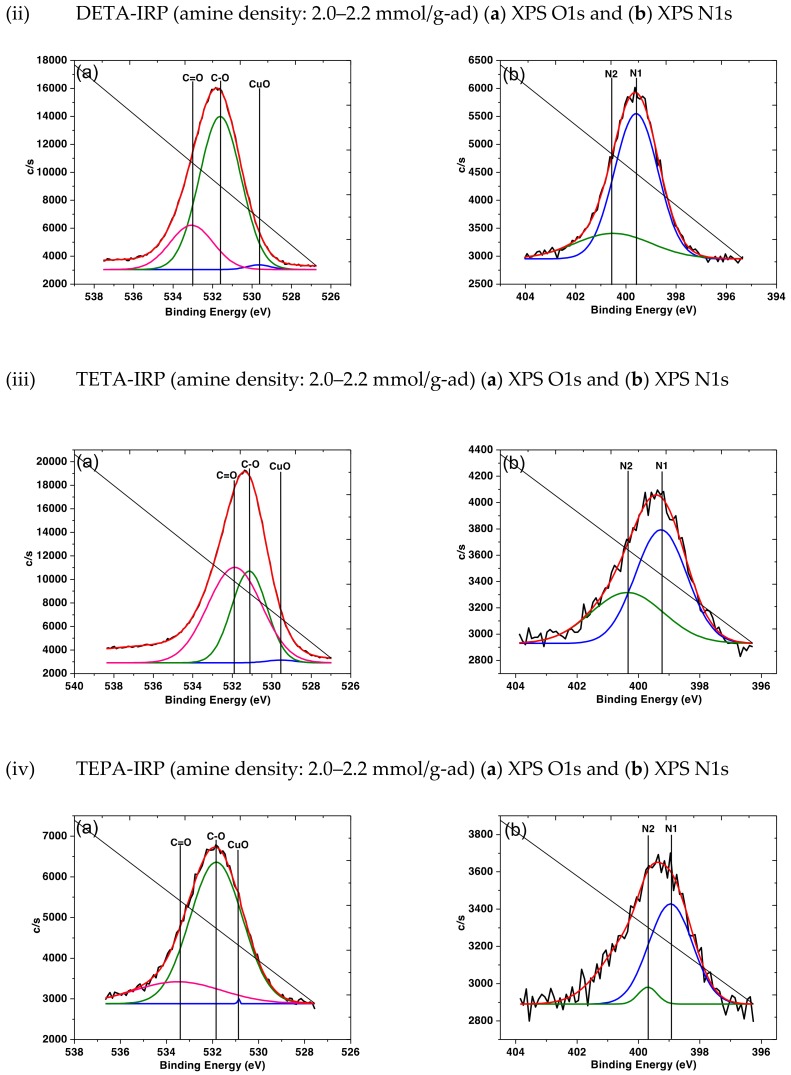
XPS spectra of (**i**) EDA-IRP, (**ii**) DETA-IRP, (**iii**) TETA-IRP and (**iv**) TEPA-IRP

X-ray photoelectron spectra of the IRP samples after adsorption with Cu ion was analyzed to verify the presence of metal ion into the polymer matrix. Thus, only the IRP samples subjected to XPS analysis. O1s and N1s orbitals were detected in EDA-IRP, DETA-IRP, TETA-IRP, and TEPA-IRP samples. The result obtained showed in Figure 8i–iv. Strong peak was observed at 399.48 eV and 400.37 eV in the N1s and N2s, which indicates primary and secondary amine groups of EDA, DETA, TETA, and TEPA, respectively [40]. The O1s spectrum of EDA-IRP, DETA-IRP, TETA-IRP, and TEPA-IRP samples shows the presence of oxygen bonding as such C–O and C=O at a binding energy of 531.70 and 533.22 eV, originating from the functional monomer, GMA [41]. The O1s spectrum of all IRP samples exhibits a broad distribution at higher binding energy due to the presence of oxygen bonded carbon and Cu concurrently in the IRP samples. The main peak at 531.70 eV attributes to the present of C–O along with binding energy at 530.30 eV indicates the existence of CuO. The XPS performed onto IRP samples had verify the chemical concentration of the samples after adsorption of Cu ion into polymer matrix. Conclusively, clear evidence was obtained on adsorption of Cu ions onto IRP samples which attribute to detection of CuO that allowed carbonyl groups from GMA to bond with.

The local coordination environment of metal ions can be selectively investigated with XAFS spectroscopy [42]. XAFS is also capable of determining distances, the coordination number, and the types of atoms surrounding the metal centers [43]. This XAFS information on metal system could be interpreted by fitting structurally well-characterized model complexes as a reference XAFS experimental data [44]. In this study, XAFS was used to investigate the molecular structure of surface complexes of Cu (II) adsorbed onto both amines immobilized GMA-*g*-NWF and IRP samples which had been modified with a different aliphatic amine, EDA, DETA, TETA, and TEPA, respectively. The structural parameters of both amines immobilized GMA-*g*-NWF, and IRP complexes were determined from the extended X-ray absorption fine structure (EXAFS) spectra and have been reported in Table 5. The main aim of this analysis was to determine the structural changes in metal complexes after radiation crosslinking process with DVB along with different aliphatic polyamine. Based on the fitting of EXAFS spectra, the Cu–O (at ~4.2 Å) and Cu-N (at ~2.0 Å) shells were observed in the Cu (II) adsorbed onto for both amine immobilized GMA-*g*-NWF and IRP samples. This observation indicating the formation of inner-sphere complexes on amine immobilized GMA-*g*-NWF and IRP samples surfaces. Different fitting values obtained for ΔR indicates a similar path for both amines immobilized GMA-*g*-NWF and IRP complexes, Cu–O and Cu–N. Moreover, the ΔR defined for the two axial Cu–O and Cu–N paths for all the amine immobilized GMA-*g*-NWF and IRP complexes. It is clear that Cu–O and Cu–N paths have effective contributions to the experimental EXAFS data. This finding agrees with XPS results of IRP samples as discussed previously. The outcome from this study supported by another study which reported on EXAFS investigation on Cu(II) complexes [10].

## 4. Conclusions

Amine immobilized GMA-*g*-NWF were successfully produced via pre-radiation grafting technique which followed by functionalization process with several aliphatic amines. The aliphatic amine concentration had imposed a significant effect on the AD yield during the functionalization process. However, the AD reduces as the aliphatic amine chain grows more extended, from EDA to TETA. The addition molecular weight and bulky structure further attribute steric hindrance which obstructs the corporation of amine group onto GMA. The similar trend observed in all other experimental condition which had been investigated. The AD increases linearly as the concentration of amine increases. The differences in amine immobilized GMA-*g*-NWF and IRP samples were extinguished by using various characterization tools. The structural coordination of GMA-*g*-NWF and IRP samples were analyzed with XPS and EXAFS spectroscopy. The complexation of the Cu ion with the prepared adsorbent takes place by the binding of Cu–O. The structural coordination of IRP samples remains the same even after the crosslinking process. The adsorptive capacity and selectively of both amine immobilized GMA-*g*-NWF and IRP samples were compared at similar experimental conditions. The IRP samples had exhibited superior adsorptive capacity and selectivity in comparison to amine immobilized GMA-*g*-NWF. The selectivity of amine immobilized GMA-*g*-NWF was greatly enhanced by associating ion reorganization technique. However, the selectivity of IRP samples reduces slightly as the aliphatic amines change form EDA to longer molecules chain of TEPA. This study particularly proves the correlation between aliphatic amine chain length with selectivity towards dedicate metal ion. The aliphatic amine with short chain gives higher selectivity in comparison to those aliphatic amines that have a long chain. In the future, the adsorption capacity and selectivity of IRP samples should be tested in multiple metal systems to evaluate the adsorptive properties comprehensively.

## Figures and Tables

**Table 1:** Effect of different amine concentration on amine density at 60 °C for 2 h, *D*_g_ = 107.42%.

Aliphatic Amines	50:50	60:40	70:30
	AD (mmol/g^−ad^)	AD (mmol/g^−ad^)	AD (mmol/g^−ad^)
EDA	5.12	4.12	3.80
DETA	4.06	3.42	3.39
TETA	3.04	2.98	3.08
TEPA	2.56	2.59	2.58

**Table 2:** Effect of different grafting yield of GMA-*g*-NWF on amine density at 60 °C for 2 h, 70% amine: 30% isopropanol.

	100%		150%		200%		250%	
Aliphatic Amines	Dg (%)	AD (mmol/g^−ad^)	Dg (%)	AD (mmol/g^−ad^)	Dg (%)	AD (mmol/g^−ad^)	Dg (%)	AD (mmol/g^−ad^)
**EDA**	105.13	3.35	151.46	2.36	205.58	2.34	251.32	1.64
**DETA**		3.54		3.03		2.53		1.92
**TETA**		4.01		3.43		2.70		2.00
**TEPA**		4.20		3.64		3.09		2.26

**Table 3:** Effect of different reaction temperatures on amine density at for 2 h, 70% amine: 30% isopropanol, *D*_g_ = 105.93%.

Aliphatic Amines	60 °C	70 °C	80 °C
	AD (mmol/g^−ad^)	AD (mmol/g^−ad^)	AD (mmol/g^−ad^)
EDA	3.85	3.90	3.93
DETA	3.31	3.50	3.52
TETA	2.97	3.25	3.28
TEPA	2.49	2.65	2.71

**Table 4:** Effect of reaction times on the amine density at for 70% amine: 30% isopropanol, 60 °C *D*_g_ = 104.86%.

Time (min)	EDA	DETA	TETA	TEPA
	AD (mmol/g^−ad^)	AD (mmol/g^−ad^)	AD (mmol/g^−ad^)	AD (mmol/g^−ad^)
15	2.89	2.41	1.61	1.21
30	3.80	3.16	2.64	2.15
45	3.82	2.97	2.52	2.36
60	3.86	3.11	2.56	2.36
90	3.87	3.20	2.59	2.41
120	3.91	3.16	2.61	2.51

**Table 5:** Structural parameters of both amines immobilized GMA-*g*-NWF and IRP complexes from EXAFS spectra.

Sample	Shell	N	R (Å)	σ^2^ (Å^2^)	E_0_ (eV)
**EDA-IRP**	Cu–O	4.000	4.061	0.029	7.622
	Cu–N	6.000	2.014	0.008	3.501
**DETA-IRP**	Cu–O	4.000	4.085	0.004	7.100
	Cu–N	6.000	2.038	0.012	3.749
**TETA-IRP**	Cu–O	4.000	4.001	0.020	7.600
	Cu–N	6.000	2.014	0.010	2.652
**TEPA-IRP**	Cu–O	4.000	3.999	0.025	7.501
	Cu–N	6.000	2.038	0.014	4.709
**EDA-GMA-g-NWF**	Cu–O	4.000	4.205	0.004	4.045
	Cu–N	4.000	1.999	0.003	4.058
**DETA-GMA-g-NWF**	Cu–O	4.000	4.218	0.004	4.124
	Cu–N	4.000	1.993	0.003	3.882
**TETA-GMA-g-NWF**	Cu–O	4.000	4.253	0.004	4.253
	Cu–N	4.000	1.973	0.005	1.657
**TEPA-GMA-g-NWF**	Cu–O	4.000	4.240	0.007	4.240
	Cu–N	4.000	1.968	0.004	0.197

## Supplementary Materials

The following are available online at https://www.mdpi.com/2073-4360/11/12/1994/s1.
