# Supplementary material for: Selectivity of Copper by Amine-Based Ion Recognition Polymer Adsorbent with Different Aliphatic Amines
Source: Polymers (Basel). 2019 Dec 2;11(12):1994. doi: 10.3390/polym11121994 (PMC6960742; doi:10.3390/polym11121994)
Supplement: Supplementary file 1 [file polymers-11-01994-s001.pdf]

polymers

MDPI

Supplementary Materials

SELECTIVITY OF COPPER BY AMINE-BASED ION RECOGNITION POLYMER ADSORBENT WITH DIFFERENT ALIPHATIC AMINES

Nor Azillah Fatimah Othman[1,*], Sarala Selambakkannu[1], Tuan Amran Tuan Abdullah[2], Hiroyuki Hoshina[3], Suchinda Sattayaporn[4], and Noriaki Seko[3,*]

[1] Radiation Processing Technology, Malaysian Nuclear Agency, Bangi, 43000, Selangor, Malaysia; azillah@nm.gov.my (N.A.F. Othman), sarala@nm.gov.my (S. Selambakkannu)
[2] Centre of Hydrogen Energy, Institute of Future Energy, Universiti Teknologi Malaysia, 81310 Johor Bahru, Johor, Malaysia; tamran@cheme.utm.my
[3] Takasaki Advanced Radiation Research Institute, Quantum Beam Science Research Directorate, 1233, Watanuki-machi, Takasaki, 370-1292 Gunma, Japan; seko.noriaki@qst.go.jp
[4] Synchrotron Light Research Institute, Nakhon Ratchasima, 30000, Thailand; suchinda@slri.or.th
* Correspondence: seko.noriaki@qst.go.jp; Tel: +81-27-346-9380

Received: date; Accepted: date; Published: date

3.3.1 Adsorption capacity of amine immobilized GMA-g-NWF and IRP samples towards Cu

Table S 1. The adsorption capacity of Cu and Pb versus time by amine immobilized GMA-g-NWF samples at experimental condition of initial Cu/Pb concentration: 10 mg/L, adsorbent dose: 0.02 g, temperature: 30 ºC, amine density: 2.0 – 2.2 mmol/g^{-ad}, stirring speed: 200 rpm and adsorbate volume: 100 ml

Time (min)	EDA GMA-g-NWF		DETA GMA-g-NWF		TETA GMA-g-NWF		TEPA GMA-g-NWF	
	Cu (mg/g)	Pb (mg/g)	Cu (mg/g)	Pb (mg/g)	Cu (mg/g)	Pb (mg/g)	Cu (mg/g)	Pb (mg/g)
15	1.60	6.52	1.93	6.69	1.57	5.08	1.19	5.33
30	2.68	8.65	2.75	9.12	2.12	6.60	1.90	5.99
60	34.85	23.31	37.94	27.79	29.89	22.13	38.22	26.21
120	36.88	27.96	38.99	32.98	29.97	25.60	39.38	29.36
240	36.89	32.95	38.99	37.29	29.99	28.76	39.39	34.96
480	36.91	33.50	39.00	38.46	29.99	29.67	39.39	38.12

Table S 2. The adsorption capacity of Cu and Pb versus time by IRP samples at the experimental condition of initial Cu/Pb concentration: 10 mg/L, adsorbent dose: 0.02 g, temperature: 30 ºC, amine density: 2.0 – 2.2 mmol/g^{-ad}, stirring speed: 200 rpm and adsorbate volume: 100 ml

Time (min)	EDA-IRP		DETA-IRP		TETA-IRP		TEPA-IRP	
	Cu (mg/g)	Pb (mg/g)	Cu (mg/g)	Pb (mg/g)	Cu (mg/g)	Pb (mg/g)	Cu (mg/g)	Pb (mg/g)
15	1.82	0.53	1.72	0.55	2.06	0.21	1.71	0.44
30	3.24	0.66	2.12	0.72	2.83	0.28	2.01	0.52
60	5.00	0.98	4.33	1.10	4.80	0.48	3.62	0.77
120	7.95	1.54	6.45	1.63	8.00	0.81	5.36	1.12
240	24.92	2.21	22.72	2.29	24.47	1.42	19.51	1.73
480	50.41	3.55	35.52	3.41	34.71	3.47	33.72	3.23

3.3.2. Adsorption mechanism

XAFS was used to investigate the molecular structure of surface complexes of Cu (II) adsorbed onto both amines immobilized GMA-g-NWF and IIP samples which had been modified with a different aliphatic amine, EDA, DETA, TETA, and TEPA, respectively. Fourier transform (FT) of EXAFS spectra obtained were exhibited in Figure 1, and Figure 2 for both amines immobilized GMA-g-NWF and IRP samples, respectively.

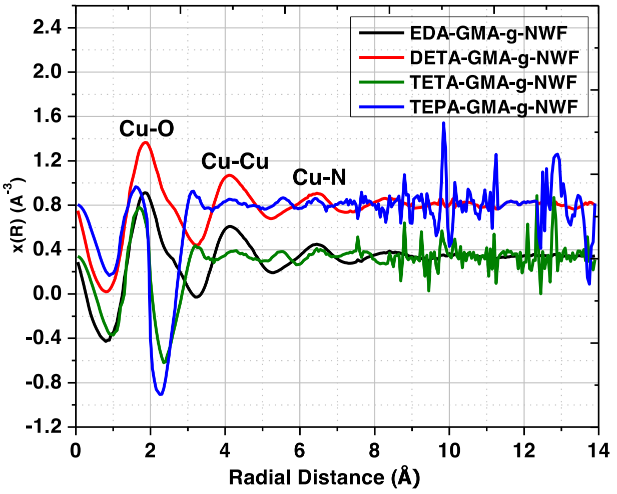

Figure S1. Fourier transform (FT) of EXAFS spectra of amine immobilized GMA-g-NWF samples which prepared at the experimental condition of 70 % amine: 30 % isopropanol, 60 ºC, Dg~100% and 2h

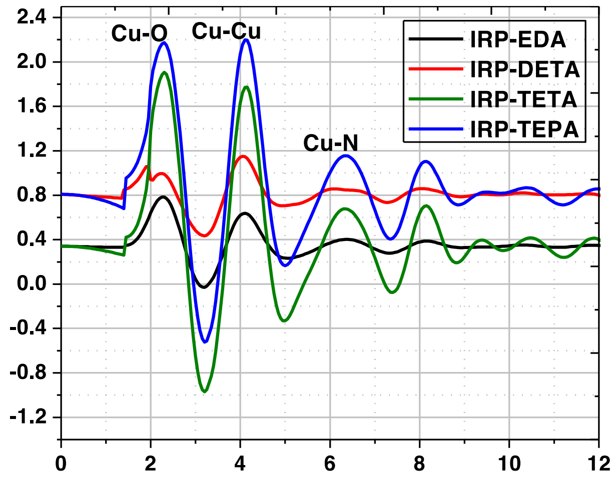

Figure S2. Fourier transform (FT) of EXAFS spectra of IRP samples which prepared at the experimental condition of 70 % amine: 30 % isopropanol, 60 ºC, Dg~100% and 2h

© 2019 by the authors. Submitted for possible open access publication under the terms and conditions of the Creative Commons Attribution (CC-BY) license (http://creativecommons.org/licenses/by/4.0/).
